# Management of sequalae of neglected septic shoulder

**DOI:** 10.4103/0019-5413.45330

**Published:** 2009

**Authors:** Uday M Pawar, Mihir Ravindra Bapat

**Affiliations:** Department of Orthopedics, P D Hinduja National Hospital, Veer Savarkar Marg, Mahim - 400 016, Maharashtra, India

**Keywords:** Sequalae septic arthritis shoulder, arthrodesis shoulder, ilizarov frame

## Abstract

Complex deformities following septic arthritis of the shoulder in infancy are mild and therefore rarely reported. A 12 year old girl presented with shortening of upper extremity right side, with dislocation of shoulder and with entire extremity rotated to 180 degrees. The palm faced posteriorly and the olecranon anteriorly. Arthrodesis of shoulder and unifocal lengthening of humerus was achieved with three 4 mm cannulated cancellous screws and an ilizarov frame. A lengthening of 9 centimeters was achieved and regenerate healed at 12 months. At 10 years follow-up she is able to perfom her activities of daily living.

## INTRODUCTION

Complex deformities following septic arthritis of the shoulder in infancy are mild and therefore rarely reported. Deformities are often combinations of various degrees of shoulder dysplasia, progressive humeral shortening, angular deformities of the humerus and subluxation of the glenohumeral joint or rarely dislocations. Even the most unsightly deformities may not produce severe functional limitations and treatment is often sought for cosmetic improvement. Guidelines for management of these complex problems are few and each case poses a challenge to the surgeon.

We report the successful management of a rare delayed presentation of septic shoulder sequelae in a pre-adolescent girl who presented with a combination of global instability of the shoulder with progressive humeral shortening.

## CASE REPORT

A 12-year-old girl presented with a complex deformity with progressive shortening involving her right dominant upper extremity. The mother gave a history of hospitalization for fever and pain in the right upper limb at 6 months of age. There was no history of trauma or surgery on the shoulder joint. The child had adapted to carry out the daily chores with her left hand. She had stopped schooling, as her writing speed and clarity with her left hand was impaired. Activities of personal hygiene were managed well.

**Figure 1:**
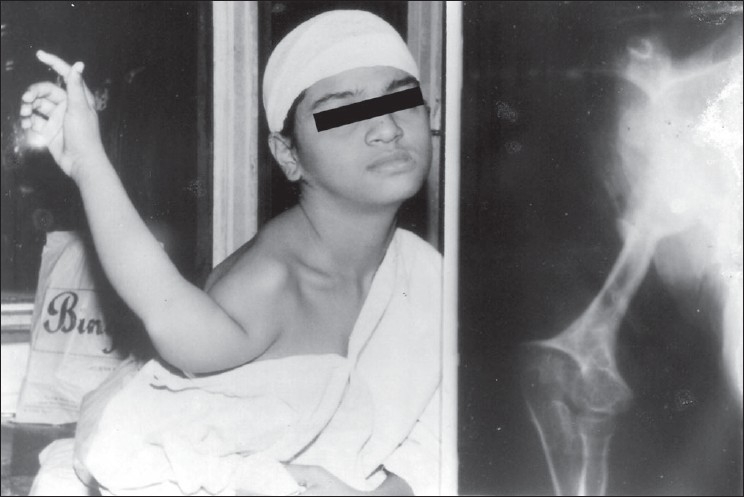
Preoperative clinical picture and roentgenogram (anteroposterior view) of humerus showing the deformity

A thorough examination ruled out congenital deformity. There was a scar along the posterior axillary fold healed with secondary intention. The affected humerus was shortened by 16 cm (right humerus measured 8 cm, left 24 cm) without angular deformities. The shoulder was dislocated with the head situated inferior and posterior to the glenoid cavity rotating the entire extremity through almost 180°. The palm facing posteriorly and the olecranon anteriorly. Attempts to position the arm across the chest actively exaggerated the deformity [Figure 1].

The humeral head was enlarged but easily reducible. This significantly improved the disability by placing the hand in front of the chest. The elbow joint and the hand were normal with wasting of upper limb. There was no neurovascular abnormality.

Plain roentgenograms showed a dislocated glenohumeral joint with enlarged humeral head and a small dysplastic glenoid fossa. The humeral shaft was short and thin, condyles were dysplastic, but the elbow joint was normal. There were no angular deformities.

A clinicoradiological correlation revealed two components of the deformity, instability as major cause of functional impairment and humeral shortening that led to the cosmetic deformity.

**Figure 2:**
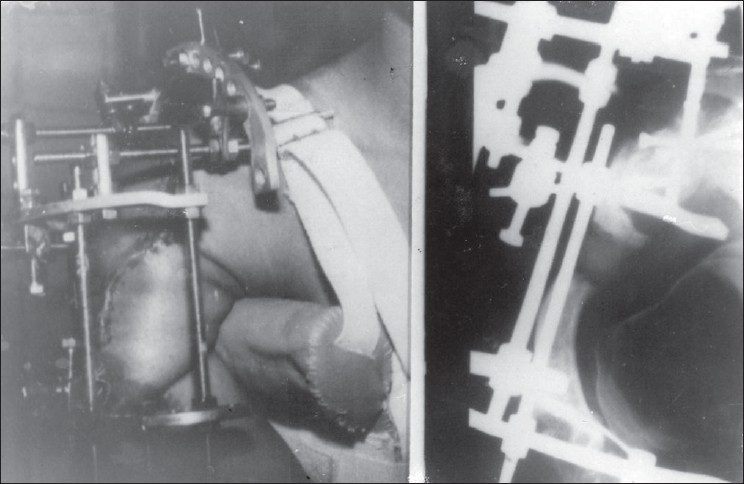
Distraction phase clinical and radiological picture (anteroposterior view) showing ilizarov fixator in situ showing progression of lengthening phase

We decided to correct the deformity by combining shoulder arthrodesis with humeral lengthening as a single-stage procedure. The obstacles were dysplasia of the shoulder that decreased the area of contact at the glenohumeral joint, small girth of the humeral shaft, quality of bone, type of fixation, and the soft tissue constraints during lengthening. We formulated the following surgical plan. The patient was kept in the lateral position. Arthrodesis of the shoulder was performed through the deltoid splitting approach, with three 4 mm cannulated cancellous screws (two across the joint and one to transfix the acromion). An Ilizarov frame was constructed to provide unifocal lengthening of the humerus and stabilization of the arthrodesis site. Five 3.5 mm Schanz pins were inserted, two in the scapular spine, two in the proximal humerus, and one in the distal humerus. The distal humeral fixation was augmented with two 1.5 mm wires. A frame was assembled with 180° half-ring for the scapula and the proximal humerus and a 5\8 ring for the distal humerus. Osteotomy was performed just distal to the deltoid tubercle [Figure 2].

The elbow range of movement exercises were encouraged as soon as the pain subsided. The distraction was commenced one week after the surgery at the rate of 0.25 mm four times a day. Roentgenograms were taken twice weekly for one month and then once monthly for three months. Uniform column of regenerate was observed. There was no distraction at the arthrodesis site, which went on to good fusion. A lengthening of 9 cm was achieved [Figure 2]. There was no neurovascular compromise and the elbow movement was maintained with limitation of terminal flexion. Problems encountered were superficial pin tract infection in the distal humeral pins, pain due to soft tissue stretching, and angular deformity of the regenerate. The distraction phase was discontinued after 14 weeks because of formation of a cyst in the regenerate with thinning of the bone. The consolidation phase lasted for further eight months, till corticalization of the regenerate and healing of the cyst was observed. The frame was then removed and a shoulder spica given for three months.

## RESULTS

**Figure 3:**
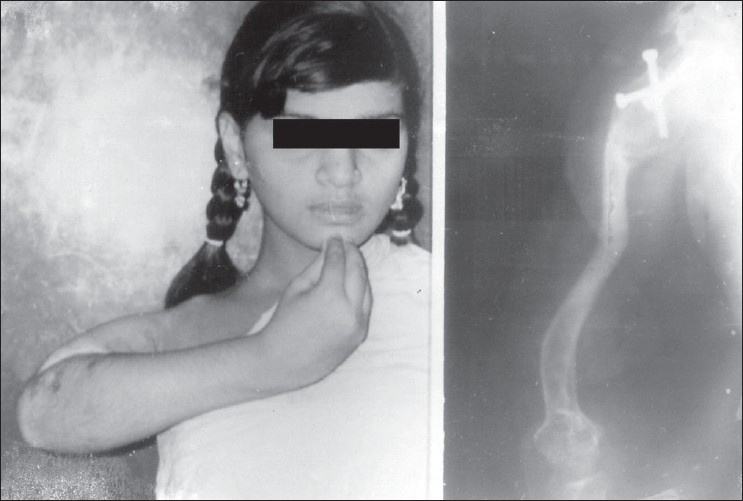
Postoperative clinical and radiological picture (anteroposterior view) after 16 months showing function of upper limb, length achieved and arthrodesis of shoulder

**Figure 4:**
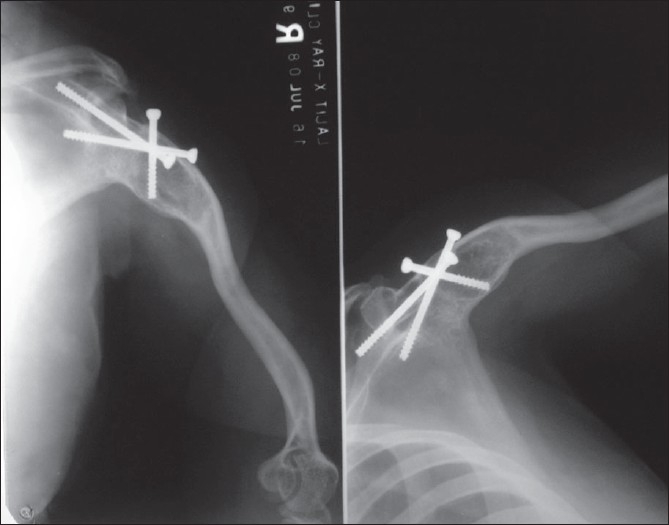
Radiographs (anteroposterior and lateral view) humerus at 10 years showing arthrodesed shoulder and length of humerus achieved

A lengthening of 9 cm was achieved and the cyst and the regenerate healed uneventfully at 12 months. The patient was advised to wear a functional humeral brace for two years and resume light, controlled activities. She started to use right hand for combing hair, scrubbing her body, cooking and writing [Figure 3]. At 10 years follow-up, the patient was able to perform her activities of daily living like lifting a bucket -full of water or objects more than 5 kg. There was no pain or discomfort at the arthrodesis site with an active abduction of 80° at the scapulothoracic joint with restricted rotations [Figure 4]. The discrepancy in length between the two arms was 7 cm. Cosmetically, the deformity was masked significantly with clothes [Figure 3].

## DISCUSSION

Complex deformities arising from neglected septic arthritis pose a challenge. These cause severe functional and cosmetic impairment in the lower limbs. However, disability in the upper limbs even with severe deformities could be minimal and hence delay the presentation. Treatment is sought more often for cosmesis.^1^,^2^ Because of rarity and variability of presentation guidelines for treatment are few. Restoration of function should be primary aim of correcting these deformities and cosmetic improvement should never be achieved at the cost of function.^3^ Ilizarov fixator by the virtue of its versatility helps in addressing several problems during the course of treatment. Humeral lengthening of 6–8 cm can be achieved safely. Correction of deformities can proceed alongside lengthening with added advantage of minimizing soft tissue and neurovascular complications.^3^ The lengthening should be carried out only till the point where a normal elbow range of movement can be maintained and good uniform column of regenerate is seen. Staged lengthening after completion of growth can be considered for severe shortening. Shoulder arthrodesis for refractory shoulder instability in children is supported in literature.^4^ Children adapt to the functional limitations imposed by the arthrodesis well. The incidence of stress fractures in the humeral shaft is less when such arthrodesis is done for paralytic shoulders.^5^ The use of an AO plate for the arthrodesis has gained popularity. In our case, the instability was the cause of the functional disability. The use of cannulated cancellous screws ensured a stable arthrodesis and provided space on the scapular spine and humerus to apply the fixator. This would not be possible if a plate was used for arthrodesis.
